# Supplementary material for: Attentional selection and communication through coherence: Scope and limitations
Source: PLoS Comput Biol. 2024 Aug 5;20(8):e1011431. doi: 10.1371/journal.pcbi.1011431 (PMC11326628; doi:10.1371/journal.pcbi.1011431)
Supplement: Attachment — Submitted filename: PLOS_CB_response to reviewers.pdf [file pcbi.1011431.s002.pdf]

**We thank the reviewers for their useful comments on the previous version of the ms. We hope that our revisions have adequately answered their reservations. They certainly have inspired us to improve the ms.**

**Responses to reviewers in bold:**

Reviewer #1:

The authors predict a very dominant role of pulvinar alpha oscillations in organizing the gamma oscillations of connected areas.

**We note that our ms is an attempt to evaluate an extended model of a mechanism for producing synchronized gamma oscillations in cortex though input of alpha oscillations from the pulvinar. We are not committed to the ultimate correctness of this model of a mechanism; predictions made are predictions of the elaborated model, which we offer as an inspiration for further development.**

However, there is little evidence for such prominent coherent alpha oscillatory activity between areas representing an attended stimulus. The proposed mechanism requires substantial alpha input to both areas representing the selected signal (with phase offset). The authors do not discuss or even mention the plentiful experimental findings, lacking coherence in alpha between two populations engaged in processing the relevant stimulus, although they should receive common pulvinar alpha (in attend in; examples: Bosman 2012 (doi: 10.1016/j.neuron.2012.06.037), Grothe et al., 2012 (DOI: https://doi.org/10.1523/JNEUROSCI.0890-12.2012), Gregoriou et al., 2009 (DOI: 10.1016/S0079-6123(09)17603-3), to name a few).

**We have addressed this comment in an expanded discussion section on the role of alpha oscillations in attention. This is a nuanced topic, and we go to considerable lengths there to present and evaluate all of the most relevant experimental evidence regarding the role of alpha oscillations, including the mentioned papers as well as a number of additional references.**

The authors mention that alpha power is generally lower in attend-in conditions compared to attend-out conditions in EEG and MEG studies. However, there is also evidence of higher alpha in attend-out conditions without stimuli and gamma oscillations present as in the baseline of attend-in conditions (contrary to model prediction; Fries et al., 2008 (DOI:10.1523/JNEUROSCI.4499-07.2008), the contrasting evidence is neither mentioned nor discussed and is hardly explainable with their theoretical model.

**We address this issue in the aforementioned expanded discussion section.**

Apart from the not mentioned or discussed contrasting evidence (good summary: Esghaei et al., 2022 DOI: https://doi.org/10.1016/j.tins.2022.01.003), the existing literature points towards multiple potential roles low high frequent oscillation interactions, which on top seem to be different in the early visual cortex, as compared to higher visible areas (also Esghaei 2022). The

authors claim a generality for their model and outcomes (title, abstract), which is not supported by much prior research. Some predictions their model makes are even contradicted.

**We have added also a discussion of the role of PAC in the same discussion section.**

The authors elaborate on the existing model and include a possible way of incorporating selective attention. Then, the authors should also state how model-based predictions could be tested experimentally.

**At the beginning of the discussion section we have outlined a possible experiment to test the alpha-gamma models, and use that as a frame for the discussion of the role of alpha in selective attention.**

Furthermore, the authors should rewrite the existing manuscript to ensure that readers understand that their approach explains a single experimental finding from 2012, while simultaneously having problems explaining many other experimental findings. The simplified model does not support the generalization of their outcomes to selective attention and CTC (Kastner et al., 2019 attributed pulvinar theta to spatial attention), and the manuscript should be rewritten accordingly.

**The work of Kastner et al on theta (1) does not rule out a role for alpha, and (2) has to do with the moments (phase) during theta oscillations when attention can be easily switched to another focus. The recent reviews by Kastner and colleagues emphasize this dynamics role but do not repudiate the role of alpha from their earlier studies. Moreoever, another recent paper of hers (Eredath et al, 2021, now cited) also shows that pulvinar alpha oscillations are causal for synchronization of LFPs and spikes in V4 and LIP.**

Subheading: Paradox re alpha ? (don't get it)

**We have moved this section to the discussion and expanded on the role of alpha in that section.**

Citations are missing in the introduction. Example: Importantly, though recently CTC has been challenged as getting……. (by whom?)

Same for the following sentence.

**We have added citations in these two places as well as in many others.**

Reviewer #2:

In general the paper would benefit from more graphical representations of the data. Except for the model schematic and the figure from a previous paper, there is only a table for all results. A graphical representation of how the signal (enhancement), coherence and MI is affected by phase difference, connectivity strength, noise,… would

be beneficial for the reader to understand the relationships between these different variables and the different effect sizes and compare them. Similarly, the way the "signal onset effect" (ln 264), "signal onset enhancement" (ln 283) and "signal enhancement effect" (ln 284) (are they all the same?) is computed, is not entirely clear. Here, too, a graphical representation of what max and mean measures mean would be helpful. They should plot an example, perhaps the one from row 1 of their example table, and visualize the actual signals (V1 and V2), the signal time course and their readout of the max and mean signal effects.

**We have added a figure (now Fig 1) showing stochastic paths to illustrate both the paths and measures of signal effects, and the effects of a couple of variables on paths and measures, as requested. These are now used both for the discussion of the previous findings of Greenwood and Ward (2022) and for the discussion of the first couple of rows in Table 2.**

Another problem is, that the work here is not really well understood without knowledge of the previous paper. They do briefly introduce this model, but then immediately jump to the discussion of Figure 1 and the signal onset effects, which have not been introduced to the reader. Figure 1 is not really understandable without reading the previous paper. They should explain their measures and hypotheses better for the reader. If their point for Figure 1 is, that both quasi cycles and noisy sine-waves for the alpha yield similar results, they should also choose the same phase offset. If their point is, that the signal propagation is maximal, when the phase offsets match, one part of the figure would likely also be enough. Instead, a figure of the respective oscillations with the added signal and how it propagates to the other area, for example for two exemplar phase offsets could be more helpful.

**We have added the requested information in Figure 1 and also removed part A of Fig 2 so as not to confuse the reader. We hope the improved figures and discussion of them make the conclusions of the earlier paper clearer as well as help explain the findings in Table 2.**

A question that might be discussed would be, that in this model, top-down attentional selection works by inhibiting the Pulvinar population that corresponds to the unattended signal. However, in reality, there is not just one other population, but often the entire visual field, except the attended location (for spatial attention) or all neurons NOT encoding the attended feature (for feature-based attention). This would mean the higher level attentional process would need to drive all but the attentionally selected reticular populations. How could this be biologically plausibly implemented? Especially in the

case of feature-based attention (without a retinotopic map) this might be difficult to achieve.

**We added additional discussion of this point in the description of the elaborated model.**

It is unclear from the text, whether the capture of attention by a salient signal would be enough to win the TRN competition alone, or whether the reallocation of top-down bias input is additionally strictly necessary. In all cases the authors discuss bottom-up attentional capture, they also mention the reallocation of top-down attention. But could the attention be shifted also without this? How big would the signal differences need to be to win the TRN competition? The need to invoke top-down processes for all attentional shifts would seem to be a rather slow mechanism, involving the traveling of the new salient signal to frontal areas and back, before a saccade could happen to the new attended location. But perhaps this is not necessary. However, this is not clear from the manuscript.

**We have added additional discussion of this point in the description of the elaborated model. It is presently unclear, at least from fMRI studies, whether bottom-up attention shifting happens "alone" (via the cortical ventral attention network, VAN) or always in combination with the cortical dorsal attention network becoming engaged after a salient signal occurs. Some believe the VAN is really an "interrupt" network that doesn't actually "engage" attention but rather allows attention to be engaged on a salient signal. Others don't even think that "attention" is a useful concept in this context. WE did not think it useful to delve more deeply into this controversy in this ms.**

A precarious point about the plausibility of this model for attentional effects is the enormous signal-boosting effect the authors see when adding noise to the signal, even without any additional alpha inputs. Here, instead of a 10% or so boost they see with "attention", they suddenly see a more than tripling of the signal amplitude, simply by adding noise without attending. They mention this, but don't truly discuss why this large mismatch in effect size comes about, what it could mean, and/or how the level of noise relates to this.

**We agree that this is a significant issue. Noise level is a parameter in the model, and affects the amplitude of the quasi-cycles, but there is no clear direction as to its magnitude from the literature. Note that in the absence of noise the quasi-cycles quickly damp to the fixed point, so that there is no oscillation to synchronize. So some level of noise is necessary. But clearly the large effect of**

**noise compared to the effect of the phase matching alpha is a limitation of the model. Why not just add noise to any signal? But noise has other, non-useful, effects, so that won't work. Optimal, non-zero, levels of noise, as in stochastic resonance, would seem to be indicated, but that is outside the scope of our ms. We did not detect any stochastic resonance effects in the model simulations to date.**

Regarding Coherence through Connectivity: In Schneider et al. the main point is, that coherence is seen in the LFP simply through rhythmic synaptic inputs to a target area, even without an ongoing oscillation or spike-entrainment in this target area. This case is however never tested in this model, as, even without an alpha input, all cortical areas always still oscillate at the same gamma frequency, putatively responsible for a lot of the coherence they see. The discussion of this in the results is rather short compared to how much the authors highlight this point in the introduction and also the abstract. Their argumentation of this putatively being the mechanism for "unattended" information transfer needs further clarification for the reader.

**We have added additional simulations, a figure (Fig 4), and additional discussion in the Results section, to address this comment.**

The section "Onset vs total signal effects" is raising a perhaps interesting point. Yet the reader cannot judge how transient the signal-boosting effect is, without ever seeing a time-course. I reiterate my point of more graphical representations being extremely useful for the reader.

**We addressed this by adding two figures, Fig 1 and Fig 4, including additional discussion.**

For the section "Paradox re alpha", the authors should either include this in the discussion or perform actual simulations with ongoing, competing alpha and gamma oscillations in the two cortices as they suggest here. This would be testable. Otherwise, this should not be in the results section because it is purely speculative. Biophysically it seems highly unlikely, that the gamma oscillatory (likely a PING mechanism) could on a millisecond time scale (as they discuss here) switch to suddenly be alpha oscillators by changes in synaptic properties.

**We moved this section to a separate discussion section, and expanded considerably and in detail on the role of alpha oscillations, including addressing the comments of reviewer 1 in this same section.**

Similarly, their discussion of the Chen results seems misguided. There is no evidence in the field that SOM cells are involved in the generation of alpha oscillations. A highly unnatural optogenetic drive to SOM cells at 5Hz makes the circuit resonate at that frequency, however, this is not plausibly happening under naturalistic conditions. SOM cells have instead been shown to be responsible for a low gamma oscillation between about 25 and 30Hz by Chen et al. and others. Also, these experiments were done in mice, which do not show alpha oscillations with properties comparable to primates.

**We acknowledge this point – we rather intended to use the simulations by Domhof and Tiesinga as indicating that biasing interneuron activity could, in principle, momentarily change the oscillation frequencies in a cortical area. We didn't intend to argue that the mouse mechanism would operate identically in primates. We have added to the text at this point to make this clear.**
