# Supplementary material for: Attentional selection and communication through coherence: Scope and limitations
Source: PLoS Comput Biol. 2024 Aug 5;20(8):e1011431. doi: 10.1371/journal.pcbi.1011431 (PMC11326628; doi:10.1371/journal.pcbi.1011431)
Supplement: Attachment — Submitted filename: PLOS_review.docx [file pcbi.1011431.s001.docx]

The submitted article, "Attentional Selection and Communication through Coherence: Scope and Limitations," extends current models on alpha/gamma interactions to elucidate a specific experimental finding. The authors introduce a new stage, the TNR, which modulates pulvinar input to cortical areas, influencing the coherence and information transfer between these regions. The primary focus is unraveling the mechanisms behind the attentional selection of relevant signals, incorporating top-down and bottom-up attention effects.

The authors base their model on previously published models that explain experimental findings. The earlier models do not encompass an attentional selection of relevant signals, which is the central motif of the submitted work. The authors show that the model can explain both top-down and bottom-up attentional effects on behavior for a specific parameter space.

Based on their model, the authors also point towards very relevant problems when interpreting solely phase-coherence measures between two areas.

The authors predict a very dominant role of pulvinar alpha oscillations in organizing the gamma oscillations of connected areas. However, there is little evidence for such prominent coherent alpha oscillatory activity between areas representing an attended stimulus. The proposed mechanism requires substantial alpha input to both areas representing the selected signal (with phase offset). The authors do not discuss or even mention the plentiful experimental findings, lacking coherence in alpha between two populations engaged in processing the relevant stimulus, although they should receive common pulvinar alpha (in attend in; examples: Bosman 2012 (doi: 10.1016/j.neuron.2012.06.037), Grothe et al., 2012 (DOI: https://doi.org/10.1523/JNEUROSCI.0890-12.2012), Gregoriou et al., 2009 (DOI: 10.1016/S0079-6123(09)17603-3), to name a few).

The authors mention that alpha power is generally lower in attend-in conditions compared to attend-out conditions in EEG and MEG studies. However, there is also evidence of higher alpha in attend-out conditions without stimuli and gamma oscillations present as in the baseline of attend-in conditions (contrary to model prediction; Fries et al., 2008 (DOI:10.1523/JNEUROSCI.4499-07.2008), the contrasting evidence is neither mentioned nor discussed and is hardly explainable with their theoretical model.

Apart from the not mentioned or discussed contrasting evidence (good summary: Esghaei et al., 2022 DOI: https://doi.org/10.1016/j.tins.2022.01.003), the existing literature points towards multiple potential roles low high frequent oscillation interactions, which on top seem to be different in the early visual cortex, as compared to higher visible areas (also Esghaei 2022). The authors claim a generality for their model and outcomes (title, abstract), which is not supported by much prior research. Some predictions their model makes are even contradicted.

The authors elaborate on the existing model and include a possible way of incorporating selective attention. Then, the authors should also state how model-based predictions could be tested experimentally. Furthermore, the authors should rewrite the existing manuscript to ensure that readers understand that their approach explains a single experimental finding from 2012, while simultaneously having problems explaining many other experimental findings. The simplified model does not support the generalization of their outcomes to selective attention and CTC (Kastner et al., 2019 attributed pulvinar theta to spatial attention), and the manuscript should be rewritten accordingly.

Subheading: Paradox re alpha ? (don't get it)

Citations are missing in the introduction. Example: Importantly, though recently CTC has been challenged as getting……. (by whom?)

Same for the following sentence.
