# Supplementary material for: Attentional selection and communication through coherence: Scope and limitations
Source: PLoS Comput Biol. 2024 Aug 5;20(8):e1011431. doi: 10.1371/journal.pcbi.1011431 (PMC11326628; doi:10.1371/journal.pcbi.1011431)
Supplement: Attachment — Submitted filename: Response to requirements of reviewer.docx [file pcbi.1011431.s003.docx]

Response to requirements of reviewer #2 **in bold.**

The graphical depictions of the signals and the onset effects are extremely helpful for the reader. Where they depict the signal time courses (Figures 1 and 4), they could, however, label the x-axes with seconds or ms, not with samples as they have done here. That way, it would also be straightforward to see whether the oscillation that is visible is alpha or gamma, which is now only possible by checking the methods.

**We changed the axis labels on these figures as requested.**

However, I reiterate my point of potentially also plotting different outputs of the model against the input variables to get a better idea of their relationships. Mean sigoe as a function of signal strength, signal noise, connectivity strength... Many of these are discussed in writing, but a simple plot is much more directly conveying this information to the reader than extracting this information from a table or writing.

**We have added plots of “extended” (name changed from “mean”) and max sigoe, *ρ* , and MI as a function of cortical connectivity, “cortical” noise, signal strength, and signal noise. We have also added descriptions of these results in the narrative. You will note some changes in the values in Table 2 to be consistent with the results in the new figures.**

Is there any experimental evidence, that the large, transient increase in oscillatory strength at the onset of the signal also happens in experiments?

**We have not been able to locate any experimental studies focusing on this phenomenon, although it is consistent with the typical increase in neural firing at stimulus onset, followed by adaptation to a somewhat above baseline firing level. Most papers we know of only display firing rate as a function of time, rather than oscillation amplitude. A Google scholar search didn’t turn up any that we missed. The Fries et al, 2008 paper does include some difficult to interpret evidence that gamma oscillations increase in V4 shortly after stimulus onset (attend-in relative to attend-out). We commented on this in the revision. This would be an interesting prediction to make, and so we added a couple of sentences in the discussion of the “ideal” experiment.**

minor: **Thanks for pointing these out:**
line 93: "maximum" should be "maximal"

**Done.**

sentence in ln 101 ff could be rephrased for clarity

**Done.**

ln 105 "our" should be "their" to keep it third person as they started.

**OK-done.**

ln 737: "though" should be "through". **Done.**
